# Supplementary material for: Content-rich biological network constructed by mining PubMed abstracts
Source: BMC Bioinformatics. 2004 Oct 8;5:147. doi: 10.1186/1471-2105-5-147 (PMC528731; doi:10.1186/1471-2105-5-147)
Supplement: Additional File 5 — The original Chilibot query results of the term "long-term potentiation (LTP)" and 22 other terms, limiting the latest references analyzed to the years 1990, 1995, 2000, and 2004. [file 1471-2105-5-147-S5.bz2 › chilibotAdditionalFile5/ltp2004/html/ACTIN_SYNAPTOPHYSIN.html]

 


 **ACTIN** and **SYNAPTOPHYSIN** 
  
Found 121 abstracts in PubMed,  **30 abstracts were retrieved and analyzed**.  


---

 Search Google  |
 PDF files only 
|  EDU domain only 

---

**Interactive relationship** (e.g. stimulation, inhibition, etc)

**Parallel relationship** (e.g. studied together, co-existance, homology, etc.)

- These changes were accompanied by a reduction in the content of  **synaptophysin**  and  **actin**  proteins.  Ref: 11933053 J Neurosci Res, 2002
- Immunohistochemical studies revealed the neoplastic cells to be positive for antibodies for CD99 and focally for  **synaptophysin** , while keratin, chromogranin, smooth muscle  **actin** , desmin, and neurofilament protein were negative.  Ref: 12376920 Ann Diagn Pathol, 2002
- Our real time PCR analysis results indicate that mRNA expression can be detected in all brain specimens for beta  **actin** , 18S rRNA, GAPDH, and also  **synaptophysin** , a known marker for AD.  Ref: 14687679 J Neurosci Methods, 2004
- Immunohistochemistry revealed that the perivascular small cells were vimentin and focally S 100 protein positive, but were negative for glial fibrillary acid protein,  **synaptophysin** , neurofilament protein, microtubule associated protein 2, neuronal nuclei antigen, nestin, carbonic anhydrase II, myelin basic protein, CD68, factor VIII, cytokeratin, epithelial membrane antigen, alpha smooth muscle  **actin** , CD20, CD45 and CD45RO.  Ref: 12111358 Acta Neuropathol (Berl), 2002
- Choroidal wholemounts and serial sections of human donor eyes were stained with antibodies against neuronal nitric oxide synthase nNOS, vasoactive intestinal peptide VIP, tyrosine hydroxylase TH, vesicular monoaminergic transporter VMAT 2, vesicular acetylcholine transporter VAChT, neuropeptide Y NPY, substance P SP, calcitonin gene related peptide CGRP, calretinin, galanin,  **synaptophysin** , and alpha smooth muscle  **actin** .  Ref: 14744873 Invest Ophthalmol Vis Sci, 2004
- METHODS TTF 1, SP A, SP B, epithelial membrane antigen EMA, pancytokeratin AE 1 AE 3, vimentin, CK7, CK5 6, calretinin, S 100, neuron specific enolase NSE,  **synaptophysin**  Syn, chromogranin A CgA, CD 34, Factor VIII and smooth muscle  **actin**  SMA in 42 patients with PSH were examined with immunohistochemistry, while samples from 10 patients were also observed by electron microscope.  Ref: 14720435 Zhonghua Jie He He Hu Xi Za Zhi, 2003
- MAIN OUTCOME MEASURES Electron microscopy and immunohistochemical staining for vimentin, smooth muscle  **actin** , muscle specific  **actin** , desmin, S 100 protein, neuronal specific enolase, chromogranin A,  **synaptophysin** , CD34, CD117 c kit, Bcl 2, and Ki 67.  Ref: 12375611 Eur J Surg, 2002
- Stains for pituitary hormones,  **synaptophysin** , chromogranin, glial fibrillary acidic protein, cytokeratin CAM5.2, smooth muscle  **actin** , CD34, and CD68 were negative.  Ref: 12170092 Am J Surg Pathol, 2002
- Immunohistochemically, all lesions were KP1 and vimentin positive and lysozyme, S 100 protein, HMB 45, epithelial membrane antigen, cytokeratins, factor VIIIrag, CD34, muscle specific  **actin** , alpha smooth muscle  **actin** , desmin, desmin, chromogranin,  **synaptophysin** , neurofilament protein, and glial fibrillary acidic protein negative.  Ref: 12360045 Am J Surg Pathol, 2002
- METHODS Fibrotic cirrhotic livers from 22 human explants and from two rat models carbon tetrachloride intoxication, bile duct ligation were studied by means of immunohistochemistry single and double immunostaining with antibodies raised against desmin, alpha smooth muscle  **actin**  alpha SMA, glial fibrillary acidic protein GFAP, neural cell adhesion molecule N CAM,  **synaptophysin** , neurotrophins, neurotrophin receptors and alpha B crystallin ABCRYS.  Ref: 11830331 J Hepatol, 2002
- other sections were processed for immunohistological detection of chromogranin,  **synaptophysin** , vimentin and smooth muscle  **actin** .  Ref: 11785121 Pathologica, 2001
- Both endothelial and stromal cells were negative for  **synaptophysin** , S 100 protein, pankeratin, smooth muscle  **actin** , CD34, CD68, alpha1 antichymotrypsin, and lysozyme.  Ref: 12627718 Vet Pathol, 2003
- Tissue sections were immunostained with vimentin, CD34, desmin, specific smooth muscle  **actin** , S100 protein, neuron specific enolase, PGP9.5, neurofilament, bcl 2 oncoprotein,  **synaptophysin** , chromogranin A, c kit, and tau.  Ref: 11727254 Hum Pathol, 2001
- Histopathologic features were analyzed by routine HE stain and immunohistochemical stains for epithelial membrane antigen, cytokeratin, vimentin, smooth muscle  **actin** , desmin, GFAP, S 100 protein, neurofilament protein,  **synaptophysin**  and alpha feto protein.  Ref: 12564764 Neuropathology, 2002
- On immunostaining, tumor cells were positive for pancytokeratins MNF 116 and C11, epithelial membrane antigen, chromogranin and neuron specific enolase NSE, and negative for  **synaptophysin** , desmin,  **actin** , estrogen and progesterone receptors, all anterior pituitary hormones and human chorionic gonadotropin.  Ref: 12611618 Eur J Endocrinol, 2003
- On immunohistochemistry, the tumor cells were reactive to smooth muscle  **actin**  and vimentin... S 100 protein, neurofilament, desmin, epithelial membrane antigen, HMB 45, glial fibrillary acidic protein, and  **synaptophysin** .  Ref: 12923346 J Korean Med Sci, 2003
- The tumor cells of the GIST were diffusely and strongly positive for CD34 and CD117, weakly positive for smooth muscle  **actin**  5% of cells, and negative for desmin, S 100 protein,  **synaptophysin** , and cytokeratin.  Ref: 12352297 J Clin Gastroenterol, 2002
- Whole penes were removed and evaluated immunohistochemically for the nitric oxide generator neuronal nitric oxide synthase, the neuronal marker  **synaptophysin** , the endothelial marker CD31 and the smooth muscle marker alpha  **actin** .  Ref: 14665962 J Urol, 2004
- With immunohistochemistry, all the neoplastic cells strongly expressed NKI C3,  **synaptophysin** , neurone specific enolase and vimentin, some expressed smooth muscle  **actin**  and PGP 9.5, but all were negative for S100, factor XIIIa, CD34, CD56, CD57, CD68, chromogranin A, desmin, epithelial membrane antigen and von Willebrand factor.  Ref: 11590076 Oral Oncol, 2001
- These results provide a developmental timeframe for the expression of recoverin, calbindin,  **synaptophysin** , beta tubulin and  **actin** .  Ref: 14519497 Brain Res Dev Brain Res, 2003
